# How Often Do Pharmacists End up in Court? Analysis of Civil and Criminal Case Law in Poland

**DOI:** 10.3390/healthcare14172861

**Published:** 2026-09-05

**Authors:** Piotr Merks, Mark Koziol, Izabella Woźnicka, Urszula Religioni, Justyna Kaźmierczak, Tomasz Drab, Janusz Ostrowski, Paweł Kulka, Sebastian Sikorski

**Affiliations:** 1 Department of Pharmacology and Clinical Pharmacology, Faculty of Medicine, Collegium Medicum, Cardinal Stefan Wyszyński University, 01-938 Warsaw, Poland; piotrmerks@gmail.com; 2 Polish Pharmacy Practice Research Network (PPPRN), 00-867 Warsaw, Poland; justynamichner@gmail.com; 3 Pharmacy Defence Association, Birmingham B1 3EA, UK; mark@koziol.org; 4 WP Law Kancelaria Prawna Woźniccy & Partners, 02-011 Warsaw, Poland; izabella.woznicka@wplaw.pl; 5 School of Public Health, Centre of Postgraduate Medical Education (CMKP), 01-813 Warsaw, Poland; 6 Silesian Pharmaceutical Chamber, 40-637 Katowice, Poland; 7 Department of European Union Law, Maria Curie-Skłodowska University, 20-031 Lublin, Poland; tomasz.drab@mail.umcs.pl; 8 Faculty of Law and Administration, Cardinal Stefan Wyszyński University, 01-938 Warsaw, Poland

**Keywords:** pharmacists, legal liability, civil liability, criminal liability, case law, healthcare law, patient safety

## Abstract

**Background:** Pharmacists, as healthcare professionals of public trust, operate within a complex legal environment involving multiple forms of professional liability, particularly civil and criminal liability. Although these forms of responsibility are formally regulated by statutory law, the practical distinction between them remains largely dependent on judicial interpretation and case-specific circumstances. **Objective:** The aim of this study was to assess the scale and nature of judicial proceedings involving pharmacists and to identify the most common factors leading to civil and criminal liability. Additionally, the study sought to highlight the largely under-recognized phenomenon of legal actions involving pharmacists and to determine which areas of pharmacy practice generate the greatest legal risk. **Methods:** The study employed a mixed-methods design combining qualitative case law analysis with descriptive quantitative assessment. A total of 146 judicial decisions involving pharmacists were reviewed, including 43 civil and 19 criminal cases constituting the principal subject of the analysis. The examined judgments were assessed with regard to factual background, legal qualification, judicial reasoning, applied legal provisions, and type of liability imposed. Cases were additionally categorized according to the nature of the violation and the protected legal interest affected. **Results:** A total of 146 judicial decisions involving pharmacists were identified and analyzed, including 43 civil cases and 19 criminal cases. The findings demonstrate that legal proceedings involving pharmacists are not isolated events but represent a recurring phenomenon within professional practice. Civil cases were dominated by disputes related to reimbursement claims, prescription processing, and regulatory compliance, accounting for over half of all analyzed civil proceedings. In contrast, criminal cases were less frequent but displayed substantial heterogeneity, including unlawful medicine distribution, reimbursement fraud, breaches of professional duties, and other criminal offences. Importantly, only a minority of cases involved direct patient harm, while the majority arose from administrative, organizational, and regulatory obligations associated with pharmacy practice. The analysis identified prescription reimbursement procedures, medicine dispensing regulations, and pharmacy management responsibilities as the principal sources of legal risk for pharmacists. **Conclusions:** Published case law demonstrates the recurrent presence and considerable diversity of legal proceedings involving pharmacists. Most cases do not arise from direct patient harm but rather from regulatory, administrative, and organizational obligations associated with pharmacy practice. Civil proceedings are predominantly linked to reimbursement and prescription-related disputes, whereas criminal cases are less common but more diverse in nature. The results highlight the need for greater legal awareness among pharmacists, enhanced risk-management education, and stronger integration of legal competencies into undergraduate and postgraduate pharmacy training. Increased recognition of legal risks may contribute to both professional protection and improved patient safety.

## 1. Introduction

Pharmacists constitute a profession of public trust whose activities are directly associated with the protection of patient health and safety as well as the proper functioning of healthcare systems. Contemporary pharmaceutical practice extends beyond the traditional role of dispensing medicinal products and includes responsibilities related to pharmaceutical care, supervision of pharmacotherapy, preparation and verification of medications, and control of prescription processing. Recent Polish research has highlighted the increasing involvement of pharmacists in pharmaceutical care and pharmacotherapy safety, reflecting the broader transition toward more patient-centred professional practice [1]. In Poland, the professional competencies of pharmacists were further expanded in 2020 through the introduction of statutory authority to issue prescriptions under specific circumstances [2]. Empirical studies conducted after this regulatory change have confirmed a substantial increase in pharmacist prescribing and widespread use of the newly expanded prescribing authority [3,4]. The widening scope of professional responsibilities simultaneously increases the legal risks associated with pharmaceutical practice.

Due to the specific role pharmacists perform within the healthcare system, the profession is subject to a complex and multilayered legal framework encompassing both public and private law regulations [5]. These regulations govern not only the rules of professional practice but also the organization of the pharmaceutical market, reimbursement procedures, and patient safety standards. Consequently, pharmacists may incur liability under different legal regimes, particularly civil and criminal liability, each serving distinct legal functions and based on different premises of responsibility [6].

Civil liability of pharmacists is primarily compensatory in nature and is generally associated with the obligation to redress damage caused to patients, contractual partners, or public institutions. In practice, a significant proportion of civil disputes concerns relationships between pharmacies and public reimbursement institutions, particularly regarding the legality and correctness of prescription processing and reimbursement claims [7]. Criminal liability, by contrast, concerns violations of penal norms and fulfills primarily protective and preventive functions. It applies especially to conduct endangering the safety of medicinal product distribution, public health, and legally protected interests related to pharmaceutical practice [8]. Although both liability regimes may arise from similar factual circumstances, they differ substantially with regard to their legal consequences, evidentiary requirements, and standards for attributing responsibility.

Existing literature concerning pharmacists’ legal liability has focused predominantly on normative and doctrinal analyses of statutory regulations [7,9]. Comparatively little attention has been devoted to judicial practice and case law analysis, despite the fact that court decisions play a crucial role in shaping the practical boundaries of legal responsibility. International case analyses demonstrate that determining responsibility may be particularly complex in medication-related cases involving overlapping prescribing and dispensing duties of physicians and pharmacists [10]. In particular, the relationship between civil and criminal liability of pharmacists, as well as the criteria determining the legal qualification of specific conduct, remains insufficiently explored. This issue is of considerable practical importance because it directly affects the legal risk associated with the profession and influences the predictability of judicial decision-making in pharmaceutical cases.

The aim of this study is to identify the practical boundaries between civil and criminal liability of pharmacists through an analysis of judicial decisions and to determine the key factors influencing legal qualification in real-world cases. Particular attention is devoted to identifying recurring categories of disputes, dominant interpretative approaches adopted by courts, and the contextual factors influencing whether specific conduct gives rise to civil or criminal liability. The study further seeks to assess whether judicial practice provides a coherent and predictable distinction between these two forms of legal responsibility.

The analysis assumes that civil liability of pharmacists is predominantly systemic and closely connected with the functioning of reimbursement and financial control mechanisms within the healthcare system, whereas criminal liability appears less frequently but concerns violations of greater social harmfulness, particularly those affecting public health and the lawful distribution of medicinal products. Furthermore, it is assumed that the boundary between civil and criminal liability is shaped primarily by the degree of fault, the nature of the violated legal norm, and the extent of harm or risk posed to legally protected interests.

## 2. Materials and Methods

### 2.1. Study Design

This study was designed as an empirical analysis of judicial practice concerning the legal liability of pharmacists. The research adopted a mixed-methods approach combining qualitative case law analysis with elements of descriptive quantitative analysis. The methodological framework was intended to examine not only the formal legal basis of liability but also the practical manner in which courts interpret and apply civil and criminal regulations in cases involving pharmacists.

The study focused on identifying the practical boundaries between civil and criminal liability, recurring categories of disputes, and the principal factors influencing judicial qualification of pharmacists’ conduct.

### 2.2. Research Material

The research material consisted of judicial decisions involving pharmacists collected and systematized by the authors in the form of a structured analytical database. Judicial decisions were identified through a comprehensive search of the principal Polish legal databases, including Legalis (https://www.beck.pl/system-informacji-prawnej-legalis/, Access Date: 26 June 2026), LEX (https://sip.lex.pl/orzeczenia-i-pisma-urzedowe/orzeczenia-sadow/1, Access Date: 26 June 2026), the Common Courts Judgments Portal (Portal Orzeczeń Sądów Powszechnych https://orzeczenia.waw.sa.gov.pl/indices, Access Date: 26 June 2026), and the Supreme Court Judgments Database (https://www.sn.pl/pl/wyszukiwarka-orzeczen, Access Date: 26 June 2026). No predefined time restrictions were applied during the database search. The search identified potentially relevant judicial decisions issued between 1998 and 2024. However, after application of the eligibility criteria and classification of the retrieved material, the cases included in the principal civil and criminal analysis covered the period 2007–2024.

The search strategy combined terms referring to the pharmacy profession with terms referring to legal liability and judicial proceedings. Searches were performed using combinations such as “pharmacist” AND “civil liability”, “pharmacist” AND “criminal liability”, “pharmacy manager” AND “liability”, “pharmacy technician” AND “liability”, and “Pharmaceutical Law” AND “pharmacist”, together with their Polish equivalents, including “farmaceuta” AND “odpowiedzialność cywilna”, “farmaceuta” AND “odpowiedzialność karna”, “kierownik apteki” AND “odpowiedzialność”, “technik farmaceutyczny” AND “odpowiedzialność”, and “Prawo farmaceutyczne” AND “farmaceuta”. Additional searches using individual professional terms were conducted to identify judgments in which liability-related terminology did not appear in the database metadata.

The final search was conducted on 30 December 2024. Records retrieved from different databases were cross-checked using the court name, judgment date, case reference number, parties, and procedural history. Duplicate records referring to the same judicial decision were removed. Where several judgments concerned successive stages of the same proceedings, the procedural history was verified, particularly using Legalis, and only the final available judgment was retained for analysis.

**Figure 1:**
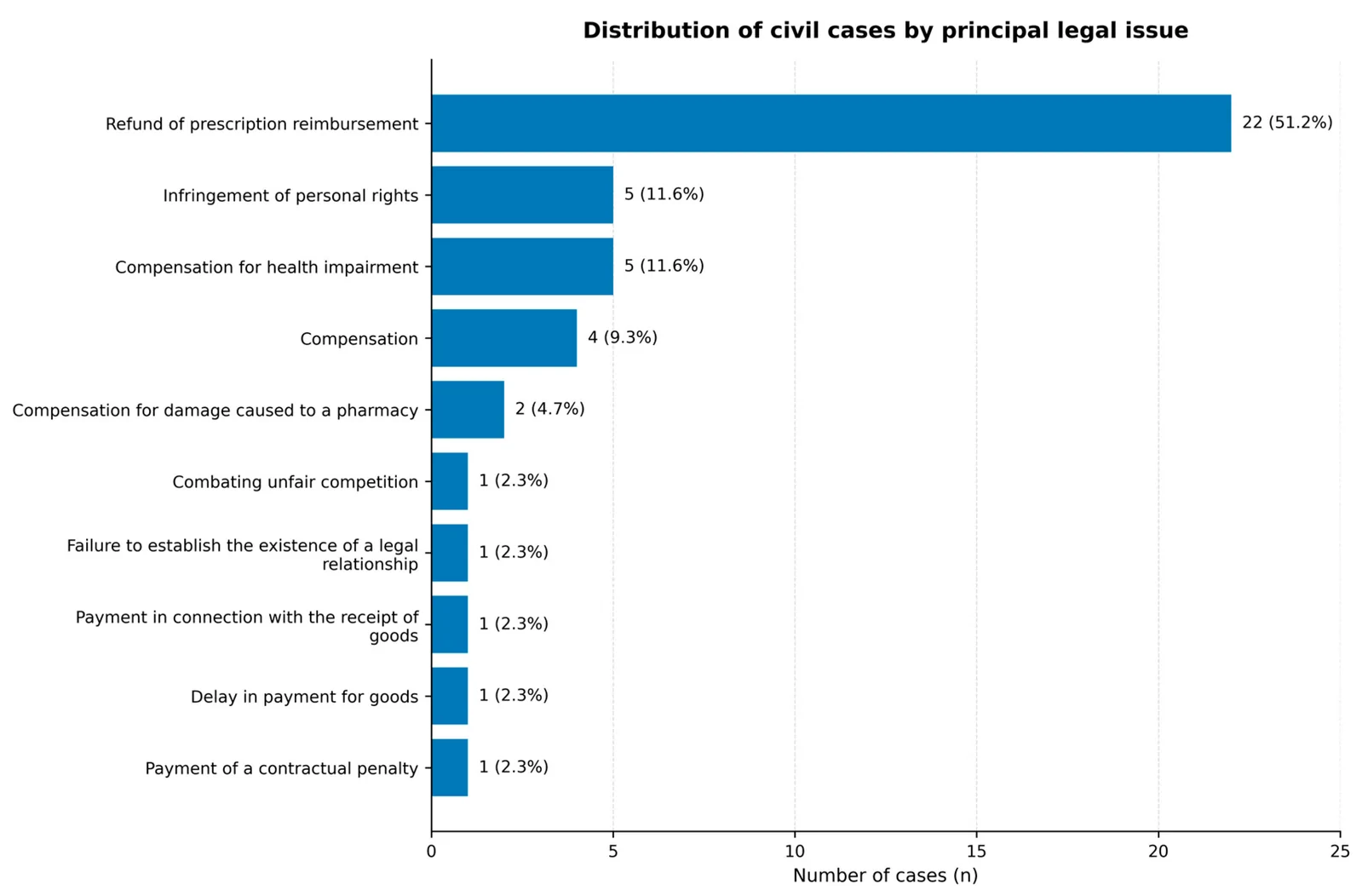
Distribution of civil cases by principal legal issue.

**Figure 2:**
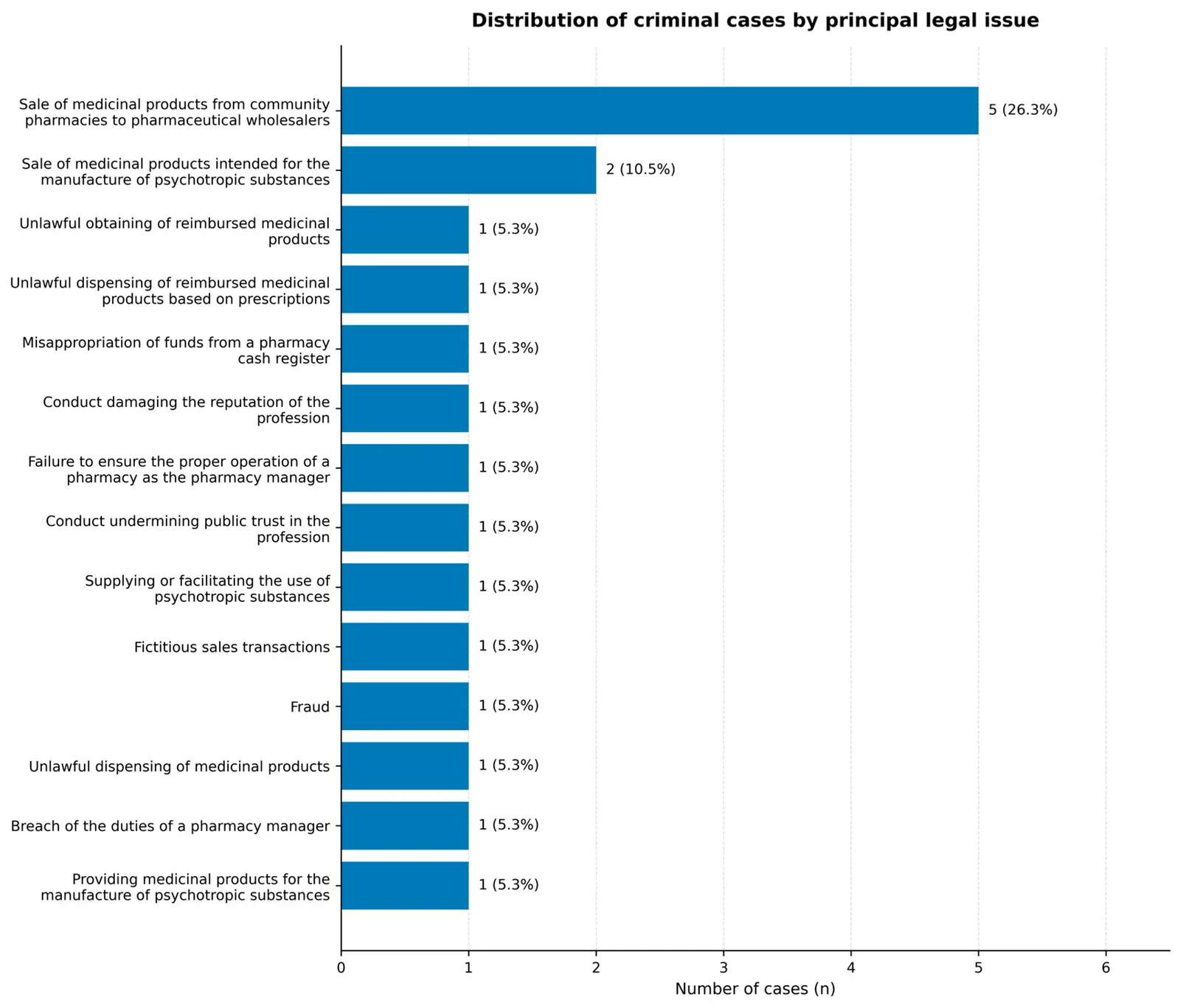
Distribution of criminal cases by principal legal issue.

**Figure 3:**
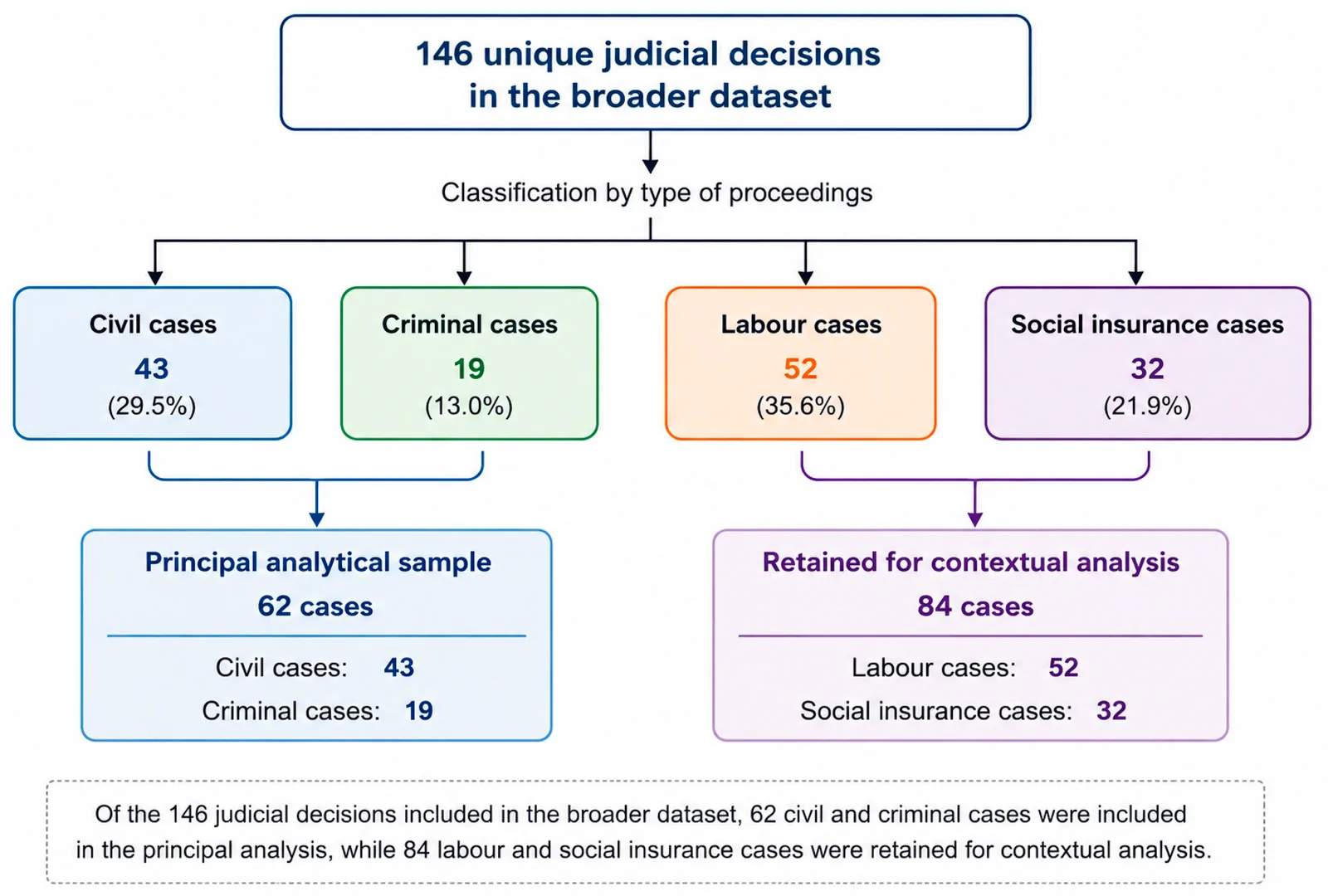
Flow diagram of the classification of judicial decisions and selection of cases for the principal analysis.

A total of 146 unique judicial decisions were included in the broader dataset following verification of the procedural history and removal of duplicate or overlapping records. These comprised 43 civil cases, 19 criminal cases, 52 labour cases, and 32 social insurance cases (Figure 1 and Figure 2). The principal analytical sample consisted of 62 cases concerning civil or criminal liability (43 civil and 19 criminal cases). The remaining 84 decisions, comprising 52 labour cases and 32 social insurance cases, were retained in the broader dataset to provide contextual information on the wider spectrum of legal proceedings involving pharmacists but were not included in the principal civil and criminal liability analysis. The process of case classification and selection for the principal analysis is presented in Figure 3.

The analyzed judgments originated from courts of various instances, including district courts, regional courts, courts of appeal, and, in selected cases, the Supreme Court. Cases were included when the professional role or conduct of a pharmacist constituted an important element of the factual background or legal assessment of the proceedings. This included cases in which pharmacists appeared as claimants, defendants, or accused persons, as well as proceedings directly evaluating professional activities performed within pharmaceutical practice. Eligible cases therefore included proceedings concerning civil liability, criminal liability, and labour or social insurance disputes related to the professional practice, employment, or legal status of pharmacists.

All judgments were initially coded and classified by IW using a predefined coding framework developed before the qualitative analysis. The coding was subsequently verified by PM. The coding variables included the type of proceedings, subject matter of the dispute, principal legal issue addressed by the court, court level, procedural role of the pharmacist, and judicial outcome. Classification was based on the factual background, legal reasoning, and operative part of each judgment rather than solely on database metadata or case titles. Where the initial classification was ambiguous, IW and PM reviewed the relevant judgment and procedural history and resolved the classification by consensus. No formal statistical measure of inter-rater agreement was calculated. The principal legal-issue category was defined as the legal question that constituted the dominant subject of the court’s assessment and was most directly related to the pharmacist’s professional activity or legal position. Civil cases included disputes concerning compensation, reimbursement, contractual or professional liability, and protection of personal rights. Criminal cases included proceedings in which criminal responsibility for conduct connected with pharmaceutical practice was assessed. Labour cases concerned employment relationships and employment-related rights or obligations, while social insurance cases concerned entitlement to, or calculation of, benefits arising under the social insurance system.

To support the qualitative analysis, the authors also prepared a supplementary statistical dataset containing classifications of cases according to the type of liability, subject matter of the dispute, dominant legal issue addressed by the court, court level, and judicial outcome.

### 2.3. Analytical Procedure

The analytical process was conducted in several stages. Initially, all collected judicial decisions underwent preliminary review and classification according to the type of proceedings. Subsequently, cases categorized as civil and criminal were subjected to detailed qualitative analysis.

For each judgment, the analysis included examination of:

- factual circumstances of the case,
- legal qualification of the conduct,
- legal provisions applied by the court,
- judicial reasoning,
- type of liability imposed,
- outcome of the proceedings.

Particular attention was devoted to identifying the criteria used by courts to distinguish between civil and criminal liability. The analysis focused especially on the role of fault, the nature of the violated legal norm, the existence of procedural or professional irregularities, and the degree of threat posed to legally protected interests, including public health and patient safety.

Within the category of civil liability, the analyzed cases most commonly concerned reimbursement disputes, irregularities in prescription processing, financial claims involving the National Health Fund, protection of personal rights, and liability for personal or financial harm. Criminal cases included proceedings related to unlawful distribution of medicinal products, breaches of professional duties, organizational negligence, and other criminal offenses involving pharmacists.

### 2.4. Data Analysis

The principal analytical method applied in the study was qualitative case law analysis, involving a detailed examination of the factual circumstances, legal reasoning, statutory provisions, and judicial outcomes of the included judgments. The objective of the qualitative analysis was to identify recurring legal problems, patterns of judicial interpretation, and differences between civil and criminal liability of pharmacists.

To complement the qualitative findings, a descriptive quantitative analysis was performed. Each judgment was coded according to predefined variables, including the year of the decision, court level, type of liability (civil or criminal), principal legal issue, legal basis, and judicial outcome. Absolute frequencies and percentages were calculated to characterize the analyzed material and to illustrate the distribution of cases across the identified categories. The quantitative results are presented in Table 1, Figure 1 and Figure 2, and Supplementary Tables S1 and S2.

The integration of qualitative and quantitative approaches enabled both an in-depth legal interpretation of individual judgments and a systematic description of the overall characteristics of the analyzed case law, thereby constituting a mixed-methods approach.

## 3. Results

### 3.1. Characteristics of the Analyzed Judgments

A total of 146 judicial decisions involving pharmacists were identified. Following the application of the eligibility criteria, 62 judgments were included in the detailed analysis, comprising 43 civil (69.4%) and 19 criminal (30.6%) cases. Although the initial search identified potentially relevant judicial decisions issued between 1998 and 2024, no cases meeting the inclusion criteria for the principal civil and criminal analysis were identified for the period 1998–2006. Consequently, the analytical period for the principal sample was 2007–2024. The analyzed judgments covered the period from 1998 to 2024 and originated from district courts, regional courts, courts of appeal, and the Supreme Court. Detailed characteristics of each judgment are presented in Supplementary Tables S1 and S2.

The descriptive quantitative analysis demonstrates that civil proceedings constituted approximately two-thirds of all included judgments, whereas criminal proceedings accounted for nearly one-third. Among the 19 criminal and professional liability cases analyzed, 11 (57.9%) resulted in a conviction or confirmation of professional liability, while 3 cases (15.8%) resulted in acquittal. In the remaining 5 cases (26.3%), the judgment was set aside and the case was remitted for reconsideration or the proceedings ended with another non-final procedural outcome. The quantitative distribution of the principal categories of civil and criminal cases is presented in Figure 1 and Figure 2 (Section 2), while the following sections provide a detailed qualitative analysis of the identified legal issues.

### 3.2. Civil Liability: Dominant Categories of Disputes

The analysis of civil cases demonstrated that the majority of disputes concerned prescription processing and reimbursement procedures within the public healthcare financing system. The most frequently identified cases involved the legality of dispensing medicinal products, formal correctness of prescriptions, and financial settlements with the National Health Fund.

A recurring issue in the analyzed judgments concerned the dispensing of medicinal products in quantities exceeding reimbursement or therapeutic limits. In the Supreme Court judgment of 20 June 2012 (I CSK 585/11), the court examined whether a pharmacist was entitled to refuse dispensing medication prescribed by a physician when the quantity exceeded the standard treatment period. The judgment emphasized the distinction between medical decision-making and pharmaceutical verification duties, indicating that determination of treatment duration remains within the physician’s competence. Similar issues were addressed in the Supreme Court judgment of 9 December 2014 (III CNP 36/13), concerning reimbursement deductions imposed by the National Health Fund following irregularities in prescription processing.

The findings also demonstrated that courts attached substantial importance to formal deficiencies in pharmaceutical and medical documentation. Cases involving missing signatures, incomplete prescription data, or illegible entries frequently resulted in challenges to reimbursement claims and financial liability of pharmacies. However, judicial practice was not entirely uniform, and several decisions indicated that purely formal irregularities did not always justify refusal of reimbursement when the factual legitimacy of dispensing remained undisputed.

A separate category of civil disputes concerned personal injury and personal rights protection. In the judgment of the District Court for Wrocław-Śródmieście of 17 May 2013 (VIII C 1383/12), the court analyzed a case involving alleged incorrect dosage information provided by a pharmacist. The claim was dismissed due to the absence of a sufficiently demonstrated causal relationship between the pharmacist’s conduct and the alleged injury. Cases concerning protection of personal rights and professional reputation were also identified, including disputes related to criticism within professional pharmaceutical organizations and allegations affecting the reputation of entities operating within the pharmaceutical market.

Overall, civil liability cases displayed a relatively homogeneous structure centered predominantly on institutional, procedural, and financial aspects of pharmaceutical practice.

### 3.3. Criminal Liability: Diversity of Legal and Factual Contexts

In contrast to civil proceedings, criminal cases involving pharmacists demonstrated substantial diversity both in terms of legal qualification and factual circumstances. The analyzed material included cases related to unlawful distribution of medicinal products, breaches of professional obligations, organizational failures in pharmacy management, as well as offenses unrelated directly to pharmaceutical practice.

The heterogeneity of criminal cases was reflected in the broad spectrum of conduct evaluated by courts, ranging from regulatory violations associated with pharmacy operations to intentional criminal acts resulting in penal liability sensu stricto. Consequently, no single dominant category of criminal cases could be identified.

An example of a case concerning pharmaceutical regulation was the Supreme Court judgment of 24 October 2017 (SDI 76/17), which addressed violations of provisions governing pharmaceutical activity and emphasized the importance of compliance with regulations concerning medicinal product distribution. Another significant example was the Supreme Court decision of 14 February 2024 (II ZK 114/22), concerning professional responsibility and the obligation to ensure proper supervision over pharmacy operations. This case demonstrated that criminal liability may also arise from organizational negligence where failures in supervision lead to violations of legally protected interests.

The analyzed material additionally included criminal offenses unrelated directly to pharmaceutical distribution, such as the judgment of the Regional Court in Piotrków Trybunalski of 19 December 2019 (III K 47/19), concerning misappropriation of property. Such cases indicate that pharmacists may incur criminal liability not only in connection with professional practice but also under general criminal law provisions applicable irrespective of professional status.

The analysis of judicial reasoning demonstrated that criminal courts attached particular significance to the degree of fault and the social harmfulness of the conduct. Determining whether the conduct was intentional, grossly negligent, or capable of creating a real threat to public health or other legally protected interests constituted a central element of judicial assessment. In this context, criminal liability fulfilled both punitive and preventive functions aimed at protecting public health and maintaining the integrity of pharmaceutical distribution systems.

### 3.4. Comparative Characteristics of Civil and Criminal Liability

Comparative analysis of civil and criminal cases revealed fundamental differences in the legal functions and practical application of both liability regimes. Civil proceedings focused predominantly on institutional and contractual relationships associated with reimbursement mechanisms and formal compliance requirements within pharmaceutical practice. Judicial assessment in such cases primarily concerned procedural correctness and financial consequences resulting from regulatory breaches.

Criminal proceedings, by contrast, involved significantly broader categories of conduct and focused on the evaluation of fault, social harmfulness, and the degree of threat posed to legally protected interests. Whereas civil liability primarily fulfilled compensatory and restitutive functions, criminal liability operated mainly as an instrument of legal protection and prevention.

Despite these differences, the findings demonstrated the existence of borderline situations in which similar factual circumstances could potentially result in either civil or criminal liability depending on contextual factors. This applied particularly to irregularities in prescription processing and breaches of professional obligations. In civil proceedings, such conduct typically resulted in financial consequences, including reimbursement claims or contractual liability. In criminal proceedings, however, analogous conduct could lead to penal liability where accompanied by a higher degree of fault, intentional conduct, gross negligence, or significant risk to public health.

## 4. Discussion

The findings of the present study demonstrate clear differences between civil and criminal liability of pharmacists, both in terms of the structure of disputes and the legal reasoning adopted by courts. Although the present analysis concerns the Polish legal system, the observed distinction between civil and criminal liability reflects broader patterns of legal accountability reported in the international literature. Across healthcare systems, pharmacists are increasingly recognized as autonomous healthcare professionals whose legal responsibility extends beyond medication dispensing to encompass patient safety, regulatory compliance, and professional decision-making [11,12,13,14,15].

One of the most significant findings of the analysis is the dominant role of disputes related to prescription processing and reimbursement procedures within the framework of civil liability [11]. Although reimbursement systems differ considerably between countries, international studies consistently identify dispensing errors, documentation deficiencies, and procedural irregularities as major sources of civil liability for pharmacists. A study conducted in the United States demonstrated that dispensing the wrong medication accounted for 36.8% of malpractice claims against pharmacists, whereas dosage-related errors represented 15.3% of claims [11]. These findings suggest that, irrespective of the legal framework, civil liability is closely associated with failures occurring during routine pharmaceutical practice, whereas the specific legal basis of such claims reflects national reimbursement and healthcare financing systems.

An important interpretative context for the obtained results is the role of regulatory and supervisory authorities. Existing studies indicate that responses to pharmaceutical errors frequently combine both educational and punitive elements, while the lack of uniform enforcement standards remains a systemic challenge [12]. Similar regulatory dilemmas have been reported across different healthcare systems, where authorities seek to balance patient protection with the promotion of a learning-oriented safety culture. This broader perspective may explain why the analyzed civil cases concentrated predominantly on formal aspects of prescription fulfilment and reimbursement procedures, which are comparatively easier to verify and enforce within institutional control mechanisms.

In contrast to civil liability, criminal liability of pharmacists demonstrated substantially greater diversity, which is likewise reflected in the available literature. Previous research suggests that serious legal violations in pharmaceutical practice are relatively infrequent but encompass a broad spectrum of conduct, ranging from regulatory breaches to intentional criminal offences. At the same time, international studies increasingly emphasize the importance of organizational and systemic factors contributing to professional errors, thereby limiting the possibility of attributing responsibility solely to individual misconduct [13]. The present findings are consistent with this perspective, as criminal proceedings generally involved circumstances extending beyond procedural non-compliance and required judicial assessment of fault, intent, and the seriousness of the resulting harm.

Particular attention should be devoted to the evolving scope of pharmacists’ professional responsibility. A study conducted in Australia demonstrated significant variation in pharmacists’ own perceptions of professional accountability, ranging from a traditional dispensing-centred model to a broader concept involving responsibility for treatment outcomes [13]. Similar developments have been observed in many healthcare systems as pharmacists assume expanded clinical roles. Such changes inevitably increase the complexity of legal accountability and may partially explain the variability observed in judicial practice, where similar factual situations may be assessed differently depending on the adopted understanding of professional duties and standards of care. Recent research on pharmacist professionalism further demonstrates that professional behaviour encompasses both technical and non-technical dimensions, including compliance with regulations, professional codes, operating procedures, and effective professional relationships [14].

The present findings should also be interpreted within the broader context of research on medication safety and healthcare quality management. Numerous studies indicate that work organization, communication failures, workload, and deficiencies in quality management systems substantially contribute to medication errors [15]. Consequently, many pharmaceutical and medical errors arise not exclusively from individual negligence but also from systemic weaknesses within healthcare organizations [12]. Evidence from hospital pharmacy settings confirms that medication errors may be associated with workload, poor communication, insufficient staff support, lack of standardization, and other organizational factors, reinforcing the importance of a systems-based interpretation of professional responsibility [16]. This systems-based approach has become a dominant paradigm in patient safety research and provides an important framework for interpreting judicial decisions concerning pharmacists’ liability. In this context, legal liability constitutes one component of a broader strategy aimed at improving medication safety and maintaining high standards of pharmaceutical care [17].

An important conclusion emerging from both the analyzed case law and the existing literature is the existence of borderline areas between civil and criminal liability [18]. Depending on the degree of fault, the seriousness of the violation, and the consequences of the conduct, similar professional irregularities may result in fundamentally different legal consequences. Comparable observations have been reported in studies concerning professional liability of healthcare practitioners, where scholars emphasize the need to consider both individual behaviour and systemic conditions when assessing professional responsibility. Although the legal mechanisms differ between jurisdictions, this distinction between compensatory and punitive functions of liability appears to represent a common feature of contemporary healthcare law.

Against the background of existing literature, the present study contributes additional value through its direct focus on judicial practice and case law analysis. Most previous studies concerning pharmacists’ liability have concentrated on medication errors, patient safety, healthcare organizations, or normative interpretation of legal regulations, whereas relatively little attention has been devoted to actual court decisions and judicial reasoning. By systematically analysing published judgments, the present study demonstrates how legal principles governing pharmacists’ professional responsibility are interpreted and applied in everyday judicial practice. Although based on Polish case law, the findings may also contribute to comparative discussions concerning pharmacists’ legal accountability in other jurisdictions by illustrating how courts distinguish between civil and criminal liability in the context of pharmaceutical practice.

The novelty of the present study lies in its comprehensive analysis of published judicial decisions concerning pharmacists’ civil and criminal liability within a single legal system. Unlike previous studies, which have primarily focused on medication errors, patient safety, or normative interpretation of legal regulations, the present study examines how legal provisions are interpreted and applied by courts in actual judicial practice. By identifying recurring patterns of judicial reasoning and distinguishing between civil and criminal liability, the study provides empirical evidence that complements the existing literature on pharmacists’ professional accountability.

Several limitations of the study should nevertheless be acknowledged. First, the analyzed dataset was purposive rather than random, which limits the possibility of generalizing the findings to the entirety of judicial practice. Second, the number of criminal cases was smaller than the number of civil cases, reflecting the structure of available case law but reducing the comparability of both categories. Third, the analysis was based exclusively on published judicial decisions without access to complete court files, which may have limited reconstruction of the full factual and evidentiary background of individual proceedings. Furthermore, despite the inclusion of descriptive quantitative elements, the study remained predominantly qualitative in nature and did not employ advanced statistical analyses. Consequently, the findings should be interpreted primarily as an empirical identification of judicial tendencies and interpretative patterns rather than as a comprehensive quantitative assessment of pharmacists’ legal liability.

It is also worth noting that the study period covers substantial legislative changes affecting pharmaceutical practice and the statutory competencies of pharmacists. Consequently, some differences observed between cases may reflect the legal and regulatory framework applicable at the time rather than stable patterns of pharmacists’ liability throughout the entire study period. In addition, no cases meeting the inclusion criteria for the principal civil and criminal analysis were identified for the period 1998–2006. This may partly reflect the more limited availability and digitization of older judicial decisions and differences in database coverage; therefore, the absence of identified cases from this period should not be interpreted as evidence that such proceedings did not occur. This should be considered when interpreting temporal comparisons and broader trends in the analyzed case law.

Despite these limitations, the study provides important insight into the practical boundaries between civil and criminal liability of pharmacists and identifies recurring judicial approaches to professional responsibility within pharmaceutical practice. While the specific legal provisions analysed are unique to the Polish legal system, many of the underlying legal and professional challenges are shared across healthcare systems. Therefore, the findings may serve as a basis for further comparative and interdisciplinary research combining legal analysis with healthcare quality, patient safety, and professional accountability perspectives.

## 5. Conclusions

The present study demonstrates that civil and criminal liability of pharmacists constitute distinct but partially overlapping legal regimes whose practical boundaries are shaped largely through judicial interpretation. Civil liability cases were predominantly associated with reimbursement procedures, prescription processing, and formal compliance requirements, whereas criminal cases were more heterogeneous and involved conduct posing greater risks to public health or the lawful distribution of medicinal products.

The findings indicate that the legal qualification of pharmacists’ conduct depends primarily on the degree of fault, the nature of the violated legal norm, and the consequences of the conduct. Similar factual circumstances may therefore result in either civil or criminal liability depending on the broader legal and factual context of the case.

The analysis further highlights the importance of procedural compliance, organizational responsibility, and professional standards in pharmaceutical practice. At the same time, the results underline the need for clearer legal guidance and greater consistency in the interpretation of regulations governing pharmacists’ professional activities.

The findings have practical implications for several stakeholder groups. For pharmacists, they highlight areas of legal risk associated with dispensing practice, prescription processing, and professional decision-making. For pharmacy managers and healthcare organizations, the identified judicial patterns may support the development of risk-management strategies, internal procedures, and professional training aimed at reducing medication errors and legal disputes. Finally, the study may contribute to future discussions on pharmaceutical regulation and professional accountability by providing empirical evidence on how legal standards are applied in judicial practice.

## Figures and Tables

**Table 1:** General characteristics of the analyzed judgments.

Characteristic	Civil Cases	Criminal Cases	Total
Number of judgments	43	19	62
Percentage	69.4%	30.6%	100%
Analytical period			2007–2024
Courts represented	District, Regional, Court of Appeal, Supreme Court	District, Regional, Court of Appeal, Supreme Court	All judicial levels
Detailed case characteristics	Supplementary Table S1	Supplementary Table S2	—
**Judicial outcome in criminal/professional liability cases**			19 (100%)
Conviction or confirmation of professional liability		11 (57.9%)	
Acquittal		3 (15.8%)	
Judgment set aside/remitted or other non-final procedural outcome		5 (26.3%)	

## Data Availability

The data supporting the findings of this study are derived from publicly available judicial decisions. Supplementary Materials provide case identifiers and key information necessary to verify the corpus of analyzed decisions, insofar as permitted by database access conditions and applicable legal requirements. Additional information is available from the corresponding author upon reasonable request.

## Supplementary Materials

The following supporting information can be downloaded at: https://www.mdpi.com/article/10.3390/healthcare14172861/s1, Table S1: Civil case law; Table S2: Criminal case law.
