# Supplementary material for: How Often Do Pharmacists End up in Court? Analysis of Civil and Criminal Case Law in Poland
Source: Healthcare (Basel). 2026 Sep 5;14(17):2861. doi: 10.3390/healthcare14172861 (PMC13565151; doi:10.3390/healthcare14172861)
Supplement: Supplementary file 1 [file healthcare-14-02861-s001.zip › healthcare-4433265-supplementary.pdf]

Table S1. Civil case law

Judgment / Court	Case No.	Case description	Legal basis	Ruling
Judgment of the Court of Appeal in Kraków- First Civil Division of 10 May 2018	I ACa 1237/17	The claimant, A.M., sought PLN 70,000 from the District Chamber of Pharmacists in K. and requested that the consequences of the infringement of her personal rights be removed by sending written apologies to all District Chambers of Pharmacists and to the claimant. The proposed apology concerned an allegedly groundless accusation that she had used a forged duplicate of her licence to practise as a pharmacist issued by the District Pharmaceutical Council in L. The court of first instance dismissed the claim, finding no infringement of the claimant's good name or professional reputation in the pharmacy community. In its letter to the Chamber in L., the defendant had not asserted that the claimant forged the document or knowingly used a forged or altered document; it merely stated that, in its view, the document had formal defects and shared those observations with other Chambers. The claimant appealed.	Art. 23, Art. 24 Civil Code	The claimant's appeal was dismissed.
Judgment of the Regional Court in Gliwice- IIFirst Civil Division Odwoławczy of 25 October 2017	III Ca 1114/17	The defendant, A.O., operated a pharmacy. After an inspection by the National Health Fund of prescriptions for reimbursed medicinal products and medical devices, 591 prescriptions were challenged and 2 prescriptions were found missing, for a total of PLN 62,881.94. The most frequent irregularities concerned the quantities of medicines dispensed, while others related to the authorization of corrections on prescriptions. The first-instance court emphasized that pharmacists are obliged to verify the correctness of prescriptions. The defendant appealed. The Regional Court accepted part of A.O.'s arguments, especially that damage to a prescription form alone could not be a sufficient basis for demanding repayment of reimbursement.	Art. 410 i 411 item 2 Civil Code, Art. 2a section 1 of the Act of 19 kwietnia 1991 r. o izbach aptekarskich and Art. 96 section 7 of the Act of 6 września 2001 r. Pharmaceutical Law, Regulation of the Minister of Health on medical prescriptions and Regulation of the Minister of Health on dispensing medicinal products and medical devices from pharmacies.	The judgment was partly amended by reducing the amount awarded in favour of the claimant against the National Health Fund.
Judgment of the Regional Court in Piotrków Trybunalski- First Civil Division of 11 March 2021	I C 1152/17	The claimants were co-owners of a pharmacy and sued a group of entities, the State Treasury represented by the Ministry of Health, and the Voivodeship Pharmaceutical Inspector. The group owned several dozen pharmacies in the voivodeship and was linked by capital and personal ties, which could qualify it as a capital group within the meaning of competition and consumer protection law. The claimants argued that the number of pharmacies run by those entities exceeded the statutory 1% threshold in the voivodeship, contrary to Pharmaceutical Law. They also alleged that state authorities had allowed distortions in the pharmaceutical market and had failed to support honest businesses.	Art. 18 of the Act on Combating Unfair Competition in conjunction with Art. 415 Code of Civil Procedure, Art. 417 Code of Civil Procedure	The claim was dismissed.
Judgment	X GC 67/16	In October 2010, several dozen pharmacists, including the defendant, sent an appeal to various	Art. 23 i 24 in conjunction with Art. 448 Civil Code	The claims were dismissed.

of the Regional Court in Łódź- Tenth Commercial Division<br>of 26 October 2017		persons and institutions concerning a capital group whose capital allegedly grew through pathological mechanisms for selling medicines. The claimants' pharmacy chain was cited as an example in that appeal. The companies sued for an order requiring the defendant to publish an apology and to pay an appropriate sum of money.		
Judgment<br>of the Court of Appeal in Gdańsk- First Civil Division<br>of 5 October 2017<br>I ACa 948/16	I ACa 948/16	The claimant company provided technical services for a medicine insurance system. It became involved in court proceedings with the Supreme Pharmaceutical Chamber concerning the assessment of medicine insurance programmes. On 16 May 2014 a notice was posted online in which the defendant, acting as President, asked pharmacists to stop meetings with the claimant's representatives at least until the court proceedings ended. The claimant sought removal of the notice and apologies, arguing that the defendant had infringed its personal right consisting in the unrestricted freedom to conduct business. The first-instance court largely accepted the claimant's position. Both parties appealed. The Court of Appeal ultimately held that the notice fell within the defendant's subjective rights as a body of the pharmacists' self-government.	Art. 43 Civil Code i Art. 24 § 1 zd. 1 i 2, in conjunction with Art. 23 Civil Code	The claimant's appeal was dismissed; the defendant's appeal was allowed by amending the first-instance judgment and dismissing the claim.
Judgment<br>of the Court of Appeal in Warsaw- Fifth Civil Division<br>of 30 March 2021	V ACa 73/21	The claimant, an association of legal persons conducting pharmacy business, sued the State Treasury seeking a declaration that no legal relationship existed under an agreement concluded to streamline administrative proceedings in cooperation with pharmacists' self-government bodies in matters concerning the ban on advertising pharmacies and pharmacy outlets. The claimant argued that the agreement was absolutely null and void. The first-instance court held that the agreement was not unlawful, relying on administrative court case-law according to which chambers of pharmacists are parties to proceedings concerning pharmacy advertising bans. The claimant appealed, and the second-instance court dismissed the appeal, among other reasons because it merely polemicized with the Regional Court's factual findings.	Art. 189 Code of Civil Procedure	The claimant's appeal was dismissed.
Judgment<br>of the Court of Appeal in Wrocław- First Civil Division<br>of 26 April 2013	I ACa 373/13	The claimant operated a pharmacy. An inspection of the Fund's expenditure revealed irregularities in 506 prescriptions. The National Health Fund demanded repayment and then set off that amount against later reimbursement receivables owed to the claimant. The claimant sued for payment, arguing that there was no formal basis for the set-off and that the irregularities were not serious enough to cast doubt on the real beneficiary of the reimbursement. The first-instance court awarded a sum to the claimant. The Fund appealed, and the Court of Appeal allowed the appeal, stressing that a pharmacist must refuse to fill prescriptions that do not meet legal requirements. Since a prohibition on	Art. 63 of the Act of 27 August 2004 on health care services, §1 i §3 Regulation of the Minister of Health z 17.05.2007 r. on reimbursed prescriptions	The defendant's appeal was allowed; the claim was dismissed and the claimant was ordered to reimburse the defendant's litigation costs.

		filling such prescriptions also means that the medicines cannot be publicly financed, the Fund was not only entitled but obliged to seek repayment.		
Judgment of the Court of Appeal in Warsaw- Fifth Civil Division of 26 September 2012	VI ACa 648/12	The claimant company operated a pharmacy. Following an inspection by the National Health Fund, a number of irregularities were found in filled prescriptions. The Fund set off the relevant amount, and the claimant sued for payment of the amount allegedly withheld without basis. The first-instance court accepted the claimant's position. The case ultimately reached the Court of Appeal, which allowed the Fund's appeal only to a minor extent, holding that the set-off was justified only for two prescriptions lacking a doctor's signature.	Art. 498 Civil Code., Art.63 ust 1 ustawy z 27.08.2004 o świadczeniach opieki zdrowotnej finansowanych ze środków publicznych	The defendant's appeal was allowed only to a minor extent; the amount due to the claimant was reduced from PLN 68,224.82 to PLN 65,810.90.
Judgment of the District Court dla Łodzi-Widzewa in Łódź- VIIFirst Civil Division of 8 September 2016	VIII C 2342/15	The defendant R.R. was a master of pharmacy employed as pharmacy manager. The company operating the pharmacy sued him for payment of a specified amount in connection with his alleged taking of certain goods. The case ended with dismissal of the claim because, although the defendant challenged the claim, he had paid the entire amount sought by the claimant by the close of the hearing.	Art. 6 Civil Code	The claim was dismissed.
Judgment of the Court of Appeal in Warsaw- Fifth Civil Division of 5 October 2012	VI ACa 856/12	The claimant operated a pharmacy. A National Health Fund inspection challenged 108 reimbursed prescriptions. The Fund demanded repayment of allegedly undue reimbursement for medicines dispensed incorrectly. The pharmacy then sued for reimbursement of those medicines. The first-instance court held that the claimant had made an error by receiving undue reimbursement. The Court of Appeal initially upheld that view. After the claimant's successful cassation appeal, the case returned to the Court of Appeal, which this time accepted the claimant's position, noting in particular that the Fund had not disputed that reimbursement had been paid and had not proved that, when making payment, it lacked knowledge of the type and quantity of medicines dispensed by the claimant.	Art. 411 item 1 Civil Code and Art. 63 section 1 of the Act on health care services financed from public funds	The claimant's appeal was allowed; the National Health Fund was ordered to pay the claimant reimbursement for medicine prices.
Judgment of the Supreme Court - Civil Chamber of 13 April 2012	I CSK 448/11	The claimant, a limited liability company, operated a pharmacy. A National Health Fund inspection established that, in 116 prescriptions, medicines had allegedly been dispensed unlawfully in quantities exceeding a three-month treatment period. The Fund demanded repayment and then set off the claim against the claimant's later reimbursement receivables. The claimant sued for reimbursement amounts not received because of the set-off. Both lower courts sided with the Fund, emphasizing the pharmacy's duty to verify quantities. The Supreme Court accepted the claimant's arguments, holding in particular that the provisions did not show that the pharmacist had grounds to refuse medicines in quantities properly prescribed by doctors.	Art. 63 of the Act of 27 August 2004 on health care services finansowanych ze środków publicznych	The cassation appeal was allowed; the challenged judgment was set aside and the Regional Court judgment was amended by awarding the claimant the relevant reimbursement amount against the National Health Fund.

Judgment of the Supreme Court - Civil Chamber of 20 June 2012	I CSK 585/11	The claimant company operated a pharmacy. A National Health Fund inspection found that, in 64 prescriptions, medicinal products had been dispensed in quantities exceeding a three-month course of treatment. The Fund set off part of the claimant's reimbursement receivables. The claimant sued for payment. The first-instance court agreed with the claimant, explaining that the doctor was fully entitled to prescribe the quantities indicated. The Court of Appeal sided with the Fund, stating that the pharmacy should have refused to dispense the medicines. The Supreme Court allowed the claimant's cassation appeal and held that determining the duration of treatment for a specific illness with a given medicine is the doctor's task, not the pharmacist's.	Art. 63 section 1 of the Act of 27 sierpnia 2004 r. o świadczeniach opieki zdrowotnej finansowanych ze środków publicznych and § 5 Rozporządzenia Ministra Zdrowia on dispensing medicinal products and medical devices from pharmacies	The claimant's cassation appeal was allowed; the defendant's appeal was dismissed.
Judgment of the Supreme Court - Civil Chamber of 29 March 2012	I CSK 332/11	The claimant operated a pharmacy. A National Health Fund inspection found that, in 108 reimbursed prescriptions, medicinal products had been dispensed in quantities exceeding a three-month course of treatment. The Fund demanded repayment and set off that claim against current reimbursement receivables. The claimant sued. The first-instance and second-instance courts sided with the Fund, holding that the pharmacy should not have dispensed quantities exceeding that limit. The Supreme Court held that the lack of factual findings concerning the actual intervals between the claimant's submission of full data and the reimbursement payments justified setting aside the judgment and remitting the case.	Art. 63 of the Act on health care services financed from public funds	The challenged judgment was set aside and the case was remitted to the Court of Appeal for re-examination.
Order of the Supreme Court - Civil Chamber of 25 June 2018	V CSK 57/18	The case concerned B.S., a master of pharmacy. He filed a counterclaim against a Voivodeship Hospital, alleging that in a letter to the Office of the Patient Rights Ombudsman in Warsaw the hospital had included several false statements defamatory to his dignity as a pharmacist. The first-instance court granted the counterclaim and ordered the hospital to publish apologies in widely read national newspapers and to pay PLN 15,000. The second-instance court considered those remedies grossly disproportionate to the manner of the infringement, reduced compensation to PLN 8,000, and ordered apologies by letter only to B.S. and the Patient Rights Ombudsman. B.S. filed a cassation appeal, which the Supreme Court refused to accept because there was no need for another ruling on the cumulation of claims under Art. 448 of the Civil Code in cases of infringement of personal rights.	Art. 448 Civil Code	The counterclaimant's cassation appeal was refused acceptance for examination.
Judgment of the Supreme Court - Civil Chamber of 9 March 2012	I CSK 216/11	The claimant operated a pharmacy as a public health-care facility. A National Health Fund inspection revealed various irregularities in prescriptions issued by doctors. The Fund set off its alleged receivables against the claimant's current reimbursement receivables. The first-instance court ruled for the claimant and awarded the full amount of allegedly wrongly set-off reimbursements. The	Art. 63 section 1 of the Act of 27 sierpnia 2004 r. o świadczeniach opieki zdrowotnej finansowanych ze środków publicznych	The claimant's cassation appeal was accepted; the second-instance judgment was set aside and the case was

		Court of Appeal dismissed the claim, reasoning that pharmacists had a duty to refuse prescriptions if irregularities were found. The Supreme Court largely sided with the claimant, holding in particular that the Fund had no right to make the set-off because its receivable did not exist.		remitted for re-examination.
Judgment of the Supreme Court - Civil Chamber of 9 December 2014	III CNP 36/13	The defendant D.K. operated a pharmacy. After a National Health Fund inspection found irregularities in a number of prescriptions, the Fund sued. The first-instance court awarded the Fund the relevant amount. The second-instance court significantly reduced it. D.K. filed a complaint seeking a declaration that the judgment was unlawful in the part awarding the amount considered justified. The Supreme Court partly accepted D.K.'s arguments because the court had breached the prohibition on adjudicating beyond the claim.	Art. 63 section 1 of the Act of 27 sierpnia 2004 r. o świadczeniach opieki zdrowotnej finansowanych ze środków publicznych	The defendant's complaint was partly allowed; the second-instance judgment was declared unlawful as to the amount awarded to the National Health Fund.
Judgment of the Supreme Court - Civil Chamber of 21 April 2010	V CSK 358/09	The claimant A.S. operated a pharmacy. A National Health Fund inspection found irregularities in 4,049 prescriptions, most often the lack of confirmation that the prescriptions had been filled. The Fund set off its alleged receivables against reimbursement amounts due to A.S. The first-instance court sided with A.S.; the second-instance court sided with the Fund, stressing that a lack of the signature of the person dispensing the medicine prevented reimbursement. The Supreme Court accepted the claimant's position because the printed name of the person dispensing the medicine, shown when the prescriptions were priced, meant that the defect had no effect on the reimbursement due.	Art. 63 of the Act of 27 sierpnia 2004 r. o świadczeniach opieki zdrowotnej finansowanych ze środków publicznych	The claimant's cassation appeal was allowed; the second-instance judgment was set aside and the defendant's appeal was dismissed.
Judgment of the Supreme Court - Civil Chamber of 14 October 2009	V CSK 103/09	The claimants C. and M. sought payment after the National Health Fund set off current reimbursement benefits against allegedly undue payments connected with prescriptions that lacked patient data or contained illegible data. Both lower courts sided with the Fund. The Supreme Court dismissed the cassation appeal because the claimants had not proved that the medicines dispensed under the challenged prescriptions reached eligible persons.	Art. 410 § 2 Civil Code	The claimants' cassation appeal was dismissed.
Judgment of the Supreme Court - Civil Chamber of 4 January 2007	V CSK 388/06	The claimant C.K. supplied medicines to a pharmacy run by A.O.K. Because she was in arrears with payment for the supplied goods, the parties concluded an assignment agreement for security under which her predecessor, the Silesian Regional Sickness Fund, was to transfer relevant benefits directly to the assignee. The Fund nevertheless paid the assignor. Both lower courts accepted the claimant's position. The Supreme Court remitted the case, emphasizing that A.O.K. could not assign an expectancy of a receivable for reimbursement of medicines and medical materials.	Art. 415, 471 i 509 Civil Code and Art. 59a of the Act on Universal Health Insurance	The defendant's cassation appeal was allowed and the case was remitted for re-examination.
Judgment	II CSKP 1158/22	The claimant pharmacy sought payment from the National Health Fund of a reimbursement amount allegedly set off without basis after an improperly	Art. 63 of the Act of 27 August 2004 on health care services,	The cassation appeal was dismissed.

of the Supreme Court - Civil Chamber of 19 January 2024		conducted inspection. The first-instance court dismissed the claim as time-barred, counting the three-year limitation period from the request for an attempted settlement. The Court of Appeal sided with the claimant on the beginning of the limitation period. The claimant nevertheless sought a substantive ruling from the Supreme Court, which did not accept the claimant's arguments, pointing in particular to the lack of evidence that the Fund knew it was making undue reimbursement.	Art. 410 i 411 Civil Code	
Judgment of the Regional Court in Szczecin- IFirst Civil Division Odwoławczy of 14 March 2018	II Ca 1139/17	The claimant concluded an agreement with the National Health Fund for dispensing reimbursed medicine. An inspection found irregularities in 15 prescriptions. The Fund called on the claimant's pharmacy manager to return the allegedly undue reimbursement and then set off its receivables against reimbursement under the claimant's statement. The claimant sued for payment. The District Court allowed the claim in full because the Fund's set-off statement had been made by an unauthorized person. The Regional Court largely upheld that ruling, finding that because the pharmacy had not breached the rules on dispensing medicines on prescriptions (dispensing more than a three-month quantity is permitted if prescribed by the doctor), the Fund did not have an undisputed receivable.	Art. 410 i 471 Civil Code and Art. 34 of the Act on health care services financed from public funds	The defendant's appeal was largely dismissed.
Judgment of the Regional Court in Wrocław- IFirst Civil Division Odwoławczy of 18 October 2017	II Ca 295/17	The claimant operated an open-access pharmacy and had an agreement for dispensing reimbursed medicine. An inspection of prescription performance found several irregularities in 144 prescriptions, and a contractual penalty was charged. The claimant sued for payment of reimbursement benefits and return of the allegedly unjustified penalty. The first-instance court dismissed the claim. On appeal, the Regional Court partly accepted the claimant's arguments but dismissed the claim for prescriptions under which the pharmacist dispensed a quantity exceeding a 90-day course, holding that such dispensing constituted an unauthorized abuse in the management of public funds and could justify refusal of reimbursement.	§ 8 section 1 item 3 Regulation of the Minister of Health z dnia 17 maja 2007 on medical prescriptions	The claimant's appeal was partly allowed.
Judgment of the Regional Court in Kielce- IFirst Civil Division Odwoławczy of 10 May 2019	II Ca 369/19	The claimant A.S. concluded an agreement with the National Health Fund for dispensing reimbursed medicine. Because of an alleged breach, the Fund demanded a contractual penalty, which she paid only to avoid compulsory enforcement. She sought return of the payment, arguing in particular that the wording of Art. 91(1)(1) of the Pharmaceutical Law at the time of the inspection did not restrict pharmacy technicians' right to prepare medicinal products containing very potent substances. The first-instance court sided with the Fund, but the Regional Court ultimately accepted the claimant's position.	Art. 91 section 1 item 1 Pharmaceutical Law and Art. 405 Civil Code in conjunction with Art. 410 § 1 i 2 i Art. 411 item 1 Civil Code	The claimant's appeal was allowed in the predominant part.

Judgment of the Court of Appeal in Lublin- First Civil Division of 29 October 2015	I ACa 335/15	The claimant M.K. worked in a pharmacy as a pharmacy technician. She was hit by a car on a pedestrian crossing and, as a result of her injuries, became permanently disabled. Because her work required standing and frequent movement, she could no longer work as a pharmacy technician. She sought compensation for non-material harm and a monthly pension. The first-instance court partly allowed the claim but dismissed the pension claim, citing her statutory right to retirement pension. Both parties appealed. The Court of Appeal partly accepted both appeals by reducing the non-material damages while establishing her right to a pension.	Art. 445 § 1 i Art. 444 § 2 Civil Code	Both parties' appeals were partly allowed.
Judgment of the District Court for Wrocław-Śródmieście- VIIFirst Civil Division of 17 May 2013	VIII C 1383/12	The claimant H.L. began taking a medicine dispensed by a pharmacist employed by the defendant pharmacy in accordance with the dosage instructions written by that employee. After starting treatment, her physical and mental condition deteriorated significantly. It later emerged that she had taken a threefold overdose because the pharmacist had made a mistake when transcribing the dosage from the prescription. H.L., and after her death M.Z., sought compensation for the health disorder. The District Court acknowledged that the pharmacist had failed to exercise special care when dispensing medicines, but held that the claimant had not proved an adequate causal link between the mistake and H.L.'s disorder and later death.	Art. 361 §1, Art.44 §1 i Art. 445 § 1 Civil Code	The claim was dismissed.
Judgment of the Court of Appeal in Lublin- First Civil Division of 15 November 2012	I ACa 522/12	The defendant E.G. was employed by the claimant pharmacy as its manager. On behalf of her employer she concluded a service agreement and an annex. A partner of the claimant sought payment of the amount allegedly taken and removed from the company, claiming she had not been authorized to conclude the agreement. The Regional Court dismissed the claim because no provision excluded a pharmacy manager from carrying out instructions other than the tasks listed in Art. 88(5) of the Pharmaceutical Law, and because she had oral authorization from the other partner, R.G. The claimant appealed. The Court of Appeal accepted the appeal, especially the argument that the legal essence of the case had not been examined, and stated that the evidence showed E.G. signed the agreement as pharmacy manager, not as attorney-in-fact.	Art. 415 Civil Code	The claimant's appeal was allowed; the challenged judgment was set aside and the case was remitted for re-examination.
Judgment of the Regional Court in Kielce- IFirst Civil Division Odwoławczy of 14 June 2018	II Ca 370/18	P.W. was trained as a pharmacy technician. While helping to cut wood, his hand was caught by a circular saw and urgent medical intervention ended with amputation of fingers. He sought additional damages and a pension from an insurer. The proceedings indicated that he could work as a pharmacy technician provided the work did not involve compounded medicines. The first-instance court accepted the claimant's position. The	Art. 444 § 1 Civil Code	The defendant's appeal was allowed; the pension claim was dismissed.

		Regional Court allowed the defendant's appeal, holding that a pension was difficult to justify because P.W. had not actually lost earning capacity in work consistent with his qualifications.		
Judgment of the Court of Appeal in Kraków- First Civil Division of 29 January 2016	I ACa 1497/15	The claimant A.K. was in her first year of education to become a pharmacy technician. As a passenger, she was injured in an accident caused by a driver travelling 50 km/h above the speed limit. The accident resulted in impairment of her left hand and interruption of her education, creating concerns about her future career. She sought non-material damages, reimbursement of rehabilitation costs, and a declaration of the defendant's future liability. The first-instance court partly accepted the claim but found that despite the accident she could obtain appropriate vocational education. On appeal, the Court of Appeal agreed with her that the additional compensation sought was adequate given her young age and the need to cope with the accident's consequences, stressing that the injuries practically deprived her of the possibility of working as a pharmacist.	Art. 445 § 1 i Art. 444 § 1 Civil Code	The claimant's appeal was allowed.
Judgment of the Court of Appeal in Szczecin- First Civil Division of 30 June 2015	I ACa 696/14	The claimant had an agreement with the National Health Fund under which his pharmacy dispensed reimbursed medicines. Despite receiving statements, the Fund did not transfer the relevant amounts, explaining that it had made set-offs based on post-inspection recommendations. The pharmacy sued. The first-instance court accepted the claim only to a small extent, stressing that the disputed prescriptions lacked legible doctors' signatures and that pharmacists must examine prescriptions for formal correctness. The claimant appealed. The Court of Appeal allowed the appeal, holding that because benefits covered by the challenged prescriptions had been issued to eligible persons, the Fund's demand for return of reimbursement was not justified.	Art. 63 section 1 i 3 of the Act on health care services financed from public funds	The claimant's appeal was allowed; the amount awarded to the claimant as reimbursement for medicines was increased.
Judgment of the Regional Court in Olsztyn- First Civil Division	I C 397/12	The claimant A.G. was an experienced pharmacy technician. She slipped on an icy pavement for which the defendant housing cooperative was responsible. Her injuries prevented her from finding work, given that the profession of pharmacy technician requires many hours of standing. She sought damages and a pension from the cooperative. The court partly allowed the claim, while stressing that her ability to find work during the period of incapacity and her seven months of unemployment before the accident had to affect the amount of the pension.	Art. 445 § 1 in conjunction with Art. 444 § 1 Civil Code	The claim was partly allowed.
Judgment of the Court of Appeal in Kraków- First Civil Division of 17 February 2020	I ACa 670/19	M.Z. worked as a pharmacy technician. She was found guilty of an offence under Art. 286(1) of the Criminal Code involving the filling of prescriptions certifying false information. The National Health Fund sought damages from her for the loss suffered. The first-instance court found for the Fund. M.Z. appealed, arguing among other things	Art. 415 Civil Code	The defendant's appeal was dismissed.

		that her participation in the scheme was marginal and that she was unaware she was acting in concert with others. The Court of Appeal rejected those arguments, relying on her final conviction and on the absence of objections as to the value of the disadvantageous disposal of property.		
Judgment of the Court of Appeal in Łódź- First Civil Division of 8 November 2016	I ACa 513/16	In October 2010, several dozen pharmacists, including the defendant, sent an appeal to numerous persons and institutions describing a capital group whose capital allegedly grew through pathological mechanisms for selling medicines. The claimants' pharmacy chain was cited as an example. The companies sought an order requiring apologies and payment of an appropriate sum. The first-instance court rejected their claims, referring in particular to broader limits of criticism toward entrepreneurs. The claimants appealed. The Court of Appeal held the appeals unfounded because, in the circumstances, freedom of expression and criticism prevailed over the claimants' interests.	Art. 23 i 24 Civil Code	The claimants' appeals were dismissed.
Judgment of the Regional Court in Łódź- IIFirst Civil Division Odwoławczy of 6 July 2018	III Ca 652/18	The claimant operated a pharmacy and had an agreement with the National Health Fund for prescription performance and medicine reimbursement. On 1 August 2015 the existing manager I.W. informed the Fund that she was ceasing work for the employer. On 3 August 2015 M.E. signed a statement accepting the manager function; on 22 October 2015 an application to change the manager was submitted; on 24 November 2015 the Voivodeship Pharmaceutical Inspector approved M.E. as the new manager; and on 21 December 2015 an annex transferred the manager position to M.E. The Fund refused to accept prescription statements for 16-31 October 2015 because they were signed by M.E. The first-instance court held that from 1 August to 24 November 2015 the pharmacy had no manager authorized to prepare and submit statements to the Fund, and therefore could not claim reimbursement for medicines sold then. The Regional Court upheld that view, emphasizing that M.E.'s statement alone was insufficient to change the pharmacy manager.	Art. 99 section 4 a in conjunction with Art. 88 Prawa Farmaceutycznego	The claimant's appeal was dismissed.
Judgment of the Regional Court in Siedlce- First Civil Division of 10 May 2017	I C 111/11	The claimant sought a declaration that no obligation existed on the basis of post-inspection findings. The National Health Fund replied that it was entitled to inspect prescriptions. It was also established that the inspectors could not contact the pharmacy manager regarding the inspection and, when they asked whether the prescriptions had been prepared, the manager did not respond. The Regional Court dismissed the claim, emphasizing that inspectors were entitled to expect contact with the pharmacy manager and that the manager was obliged to ensure an efficient and effective inspection.	Art. 410 § 2 Civil Code	The claim was dismissed.

Judgment of the Regional Court in Lublin - First Civil Division of 19 May 2014	I C 1020/12	The defendant E.G. was employed as pharmacy manager, and her employment contract did not precisely define her duties. The employer company was run by spouses who later entered into a long conflict after their marriage broke down. M.G. sued E.G. on behalf of the company for damages allegedly caused by E.G.'s signing, without proper authorization, a service agreement with the other partner R.G., which enabled R.G. to receive the amount claimed. The Regional Court ultimately dismissed the claim, holding that no fault could be attributed to E.G.; absent contrary evidence, testimony showed that the purpose was to improve the company's functioning on the national pharmaceutical market, and the agreement was not sham because R.G. actually performed services for the company.	Art. 415 Civil Code	The claim was dismissed.
Judgment of the Regional Court in Łódź- IIFirst Civil Division Odwoławczy of 30 May 2023	III Ca 2173/21	The claimant I.K., a master of pharmacy operating an open-access pharmacy, had an agreement with the National Health Fund for dispensing reimbursed medicine. An inspection found irregularities in 133 prescriptions, namely incorrect data on medicine turnover regarding the identifier of the person filling the prescriptions. The Fund imposed a contractual penalty, which was paid with a reservation of return. I.K. sued, explaining that all prescriptions submitted for inspection bore both the signature of a pharmacy technician and her signature as a master of pharmacy, and that stamped prescriptions were dispensed by a master of pharmacy. The first-instance court accepted her position, holding that because none of the prescriptions was challenged or withdrawn, the incorrect data did not amount to undue reimbursement and the Fund had no right to impose the penalty. The Regional Court dismissed the Fund's appeal, explaining that under the agreement, incorrect data alone was insufficient for a contractual penalty unless linked to undue reimbursement.	Art. 410 i 483 § 1 Civil Code	The defendant's appeal was dismissed.
Judgment of the Court of Appeal in Katowice- First Civil Division of 5 July 2013	I ACa 164/13	The claimant J.G. was a pharmacist by education and ran her own pharmacy. In a car accident caused by a drunk driver she suffered permanent injury that deprived her of the ability to perform her favourite profession. She sought compensation for non-material harm. The first-instance court awarded PLN 100,000, taking into account amounts already paid partly in the claims settlement process and partly as compensatory payment in the criminal judgment. Both parties appealed, and the Court of Appeal found both appeals unfounded.	Art. 445 § 1 Civil Code	Both parties' appeals were dismissed.
Judgment of the Regional Court in Toruń- VIIFirst Civil Division Odwoławczy	VIII Ca 192/13	The National Health Fund inspected the defendant company for correctness of prescription performance and found irregularities which, in the Fund's view, meant that certain amounts were undue. Because the defendant paid only part of the amount, the Fund sued. The District Court allowed	Art. 410 Civil Code i Art. 63 section 1 of the Act on health care services financed from public funds	The claimant's appeal was partly allowed.

of 12 June 2013		the claim only to a small extent, holding that it is not reasonable to require a pharmacist, whenever the computer system indicates that a medicine may be dispensed, to additionally check the reimbursement list to determine whether the medicine was in fact reimbursed. The Fund appealed. The Regional Court partly allowed the appeal, noting in particular that the absence of a PESEL number on a prescription disqualified it from reimbursement.		
Judgment of the Regional Court in Warsaw- Fifth Civil Division Odwoławczy of 10 June 2011	V Ca 1057/11	The claimant operated an open-access pharmacy. After the National Health Fund set off reimbursement due to him because inspection results showed that some prescriptions were challenged for reasons including missing doctor signatures and incomplete patient addresses, he sued for the amount due. The first-instance court accepted the claim, holding that lack of a precise patient address did not prevent entitlement to reimbursement. The second-instance court upheld the Fund's appeal. The Fund then applied to the Supreme Court, which set aside the judgment and remitted the case. The Regional Court partly allowed the Fund's appeal, holding that missing doctor signatures and incomplete patient addresses excluded dispensing and reimbursement, but also stated that a pharmacist was entitled to dispense medicines under prescriptions exceeding a three-month course because they were valid in order to maintain continuity of treatment.	Art. 63 section 1 of the Act on health care services financed from public funds, Art. 410 § 2 Civil Code	The National Health Fund's appeal was partly allowed.
Judgment of the Court of Appeal in Szczecin- First Civil Division of 6 September 2018	I ACa 185/18	The claimant D.N. worked as a pharmacy technician. Her mother H.R. died in a traffic accident. The suffering caused by that event affected D.N.'s ability to perform her professional duties, and she had to take sick leave. She sought non-material damages from the insurer. The Regional Court partly allowed the claim, recognizing that her inability to work evidenced her suffering, but also finding the amount sought excessive because D.N. was an adult with her own family at the time of her mother's death. Both parties appealed, and the Court of Appeal upheld the judgment.	Art. 446 § 4 i Art. 448 Civil Code	Both parties' appeals were dismissed.
Judgment of the Regional Court in Łódź- IFirst Civil Division of 23 October 2017	II C 768/16	The claimant K.W. worked as a pharmacy technician. After a traffic accident she suffered extensive bodily injuries and sought non-material damages, damages, and a pension for reduced future prospects. After finding lack of subject-matter jurisdiction, the District Court transferred the case to the Regional Court. The Regional Court partly allowed K.W.'s claim but dismissed the pension claim, finding that although her injuries were extensive, expert opinions clearly showed that they did not deprive her of the ability to work as a pharmacy technician as before the accident.	Art. 444 i 445 Civil Code	The claim was partly allowed; non-material damages and damages were awarded, and the pension claim for reduced future prospects was dismissed.

Judgment of the Regional Court in Szczecin- IFirst Civil Division Odwoławczy of 6 September 2017	II Ca 1488/16	The claimant pharmacy, managed by master of pharmacy A.K., had an agreement with the National Health Fund for dispensing reimbursed medicine. An inspection found irregularities in 17 prescriptions resulting in no reimbursement. The Fund made set-offs. The first-instance court dismissed the pharmacy's claim, holding that irregularities such as lack of the stamp of the person preparing a compounded medicine and lack of the signature of the person dispensing a medicine could not be treated as minor defects only. The Regional Court upheld the judgment, stressing that the irregularities were correctly assessed as justifying the Fund's demand for return of reimbursement.	Art. 411 Civil Code	The claimant's appeal was dismissed.
Judgment of the Regional Court in Warsaw- Fifth Civil Division Odwoławczy of 17 May 2013	V Ca 288/13	The claimant operated an open-access pharmacy where some medicines were fully or partly reimbursed by the National Health Fund. An inspection challenged 148 prescriptions for a preparation used to treat prostate cancer because more of the medicine had been dispensed than the then applicable law permitted. The claimant challenged the protocol. The Fund set off its alleged receivables, and the case went to court. The District Court accepted the claimant's position, stressing that the pharmacist had no right to refuse to fill prescriptions in the treatment of malignant tumours because doing so could endanger patients' life and health. The second-instance court upheld that position.	Art. 498 Civil Code in conjunction with Art. 63 section 1 i 2 of the Act on health care services financed from public funds.	The National Health Fund's appeal was dismissed.

Table S2. Criminal case law

Judgment	Reference No.	Judicial outcome	Conduct concerne	Legal basis/Charge	Penalty or legal consequence
Judgment Supreme Court – Criminal Chamber of 14 July 2022	I KK 212/22	Professional liability confirmed	Breach of the obligations of a pharmacy manager	Article 88(5)(1) in conjunction with Article 92 and Article 88(4) of the Pharmaceutical Law Act of 6 September 2001	Reprimand penalty
Judgment Regional Court in Lublin – XI Criminal Appeal Division of 5 November 2019	XI Ka 798/19	Conviction	Fraud	Article 286 § 3 in conjunction with § 1 of the Penal Code; Article 286 § 1 of the Penal Code in conjunction with Article 270 § 2 and Article 11 § 2 of the Penal Code	Fine and prohibition from practicing as a pharmacist
Judgment District Court in Giżycko – II Criminal Division of 23 February 2022	II K 583/18	Conviction	Unlawful obtaining of reimbursed pharmaceutical products by misleading the National Health Fund	Article 286 § 1 of the Penal Code in conjunction with Article 107(1) of the Personal Data Protection Act of 10 May 2018 and Article 12 of the Penal Code	One year imprisonment, 150 daily fine units, prohibition from holding the position of pharmacy manager for 3 years, compensation for damage caused to the National Health Fund
Judgment Court of Appeal in Wrocław – II Criminal Division of 29 October 2014	II AKa 307/14	Acquittal	Sale of 1,600 packages of medicines with awareness that the medicines were intended for the production of a psychotropic substance	Article 18 § 3 of the Penal Code in conjunction with Article 53(2) of the Act of 29 July 2005 on Counteracting Drug Addiction	Acquittal upheld
Judgment Court of Appeal in Szczecin – II Criminal Division of 6 February 2014	II AKa 212/13	Conviction	Dispensing reimbursed medicines despite the absence of documents authorizing free fulfillment of prescriptions	Article 286 § 1 of the Penal Code in conjunction with Article 270 § 1, Article 12, and Article 11 § 2 of the Penal Code	Removal of the fine; pharmacists ordered to partially remedy the damage through appropriate payment
Judgment Regional Court in Piotrków Trybunalski – III Criminal Division of 19 December 2019	III K 47/19	Conviction	Theft of PLN 190,000 from the pharmacy cash register while working as a pharmacy technician	Article 278 § 1 of the Penal Code in conjunction with Article 12 § 1 of the Penal Code	Eight months imprisonment and a fine of 100 daily units of PLN 20 each
Decision Supreme Court – Criminal	I KK 6/18	Professional liability confirmed	Conduct capable of damaging the good name of other	Article 15 of the Pharmacist Code of Ethics	Dismissal of the cassation appeal and charging the accused with cassation proceedings costs

Chamber of 24 October 2018			members of the profession		
Judgment Supreme Court – Criminal Chamber of 11 August 2020	I KK 20/20	Acquittal	Failure, as manager, to ensure proper organization of work in the pharmacy, including dispensing large quantities of prescription medicines to healthcare providers and unreliable record-keeping	Article 88(5)(1) of the Pharmaceutical Law and others	Acquittal
Judgment Supreme Court – Criminal Chamber of 6 December 2017	SDI 93/17	Judgment set aside and remitted for reconsideration	Conducting unlawful trade in medicinal products through sales to unauthorized entities, namely pharmaceutical wholesalers and public pharmacies	Articles 87(2)(1), 72(1), and 96(1)(1) and (3) of the Pharmaceutical Law	Repeal of the contested ruling and remittal for reconsideration
Judgment Supreme Court – Criminal Chamber of 24 October 2017	SDI 76/17	Judgment set aside and remitted for reconsideration	Allowing the sale of medicinal products in short supply on the Polish market from public pharmacies to pharmaceutical wholesalers	Article 15 of the Pharmacist Code of Ethics	Repeal of the contested ruling and remittal to the Supreme Pharmaceutical Court
Judgment Supreme Court – Criminal Chamber of 29 August 2017	SDI 41/17	Judgment set aside and remitted for reconsideration	Sale of medicinal products from a pharmacy to pharmaceutical wholesalers as a pharmacy manager and co-owner	Articles 65(1), 68(1), 72(1) and (3), 74(1), 86(1) and (2), and 87(2) of the Pharmaceutical Law, and Articles 1(3) and (4), 3(2), 6(1), 15, and 25(2) of the Pharmacist Code of Ethics	Repeal of the contested ruling and remittal to the Supreme Pharmaceutical Court
Decision Supreme Court – Professional Liability Chamber of 14 February 2024	II ZK 114/22	Professional liability confirmed	Undermining trust in the pharmacist profession and the dignity of the profession through absence from the hospital pharmacy while being the only pharmacist employed there	Article 45 of the Act on Pharmaceutical Chambers of 19 April 1991 in conjunction with Article 92 of the Pharmaceutical Law and Articles 1(3), 7(3), and 14(1) of the Pharmacist Code of Ethics	Dismissal of the defense counsel's cassation appeal
Decision Supreme Court – Criminal Chamber of 20 June 2023	II KS 14/23	Case remitted for reconsideration / non-final procedural outcome	Providing or facilitating the use of a psychotropic substance	Article 58(1) of the Act of 29 July 2005 on Counteracting Drug Addiction	Dismissal of the prosecutor's complaint against the appellate judgment remitting the case for reconsideration
Decision Supreme Court – Criminal	SDI 23/18	Professional liability confirmed	Unlawful dispensing of medicinal products	Article 96(1) in conjunction with Article 88(1) sentence 1 and Article 88(5)(1) of the Pharmaceutical Law	Dismissal of the defense counsel's complaint

Chamber of 6 June 2018					
Judgment Court of Appeal in Wrocław – II Criminal Division of 20 July 2023	II AKa 184/23	Conviction	Sale of medicines outside the pharmacy distribution system enabling production of psychotropic substances	Article 18 § 3 of the Penal Code in conjunction with Article 53(2) of the Act on Counteracting Drug Addiction and related provisions	Partial allowance of the appeal; suspension of the pharmacist's professional license for 4 years upheld
Judgment District Court for Warsaw-Mokotów in Warsaw – III Criminal Division of 20 April 2021	III K 867/19	Acquittal	Violation of the prohibition on selling medicinal products from a pharmacy to a pharmaceutical wholesaler	Article 126b of the Pharmaceutical Law	Acquittal
Judgment District Court in Opole – VII Criminal Division of 1 August 2018	VII K 522/18	Conviction	Fictitious sales transactions of medicines by canceling or failing to enter them into the system and collecting money from customers	Article 286 § 1 of the Penal Code in conjunction with Article 12 of the Penal Code	8 months imprisonment suspended for 1 year and compensation for damages
Judgment Regional Court in Białystok – III Criminal Division of 31 May 2023	III K 50/23	Conviction	Violation of the prohibition on selling medicinal products from a public pharmacy to a pharmaceutical wholesaler	Article 126 of the Pharmaceutical Law	6 months imprisonment, 150 daily fine units, forfeiture of financial benefits obtained from the offense
Judgment Court of Appeal in Lublin – II Criminal Division of 31 May 2022	II AKa 318/21	Acquittal judgment set aside and case remitted for reconsideration	Providing medicines for the production of psychotropic substances	Article 258 § 1 of the Penal Code and Article 53(2) of the Act on Counteracting Drug Addiction	Repeal of the acquittal judgment and remittal for reconsideration
